# Inflammatory Endotype–associated Airway Microbiome in Chronic Obstructive Pulmonary Disease Clinical Stability and Exacerbations: A Multicohort Longitudinal Analysis

**DOI:** 10.1164/rccm.202009-3448OC

**Published:** 2021-06-15

**Authors:** Zhang Wang, Nicholas Locantore, Koirobi Haldar, Mohammadali Yavari Ramsheh, Augusta S. Beech, Wei Ma, James R. Brown, Ruth Tal-Singer, Michael R. Barer, Mona Bafadhel, Gavin C. Donaldson, Jadwiga A. Wedzicha, Dave Singh, Tom M. A. Wilkinson, Bruce E. Miller, Christopher E. Brightling

**Affiliations:** ^1^ Institute of Ecological Sciences, School of Life Sciences, South China Normal University, Guangzhou, China;; ^2^ Medical Innovation, Value Evidence and Outcomes, and; ^6^ Human Genetics, Research and Development, GlaxoSmithKline, Collegeville, Pennsylvania;; ^3^ Department of Respiratory Sciences, Institute for Lung Health, Leicester National Institute for Health Research Biomedical Research Centre, University of Leicester, Leicester, United Kingdom;; ^4^ Division of Infection, Immunity and Respiratory Medicine, School of Biological Sciences, Faculty of Biology, Medicine and Health, Manchester Academic Health Science Centre, The University of Manchester, Manchester, United Kingdom;; ^5^ Institute of Statistics and Big Data, Renmin University of China, Beijing, China;; ^7^ Chronic Obstructive Pulmonary Disease Foundation, Research Department, Washington, District of Columbia;; ^8^ Respiratory Medicine Unit, Nuffield Department of Medicine, University of Oxford, Oxford, United Kingdom;; ^9^ National Heart and Lung Institute, Imperial College London, London, United Kingdom; and; ^10^ National Institute for Health Research Southampton Respiratory Biomedical Research Unit, University Hospital Southampton National Health Service Foundation Trust, Southampton, United Kingdom

**Keywords:** chronic obstructive pulmonary disease, airway microbiome, inflammatory endotypes, unbiased clusters, cytokines

## Abstract

**Rationale:** Understanding the role of the airway microbiome in chronic obstructive pulmonary disease (COPD) inflammatory endotypes may help to develop microbiome-based diagnostic and therapeutic approaches.

**Objectives:** To understand the association of the airway microbiome with neutrophilic and eosinophilic COPD at stability and during exacerbations.

**Methods:** An integrative analysis was performed on 1,706 sputum samples collected longitudinally from 510 patients with COPD recruited at four UK sites of the BEAT-COPD (Biomarkers to Target Antibiotic and Systemic COPD), COPDMAP (Chronic Obstructive Pulmonary Disease Medical Research Council/Association of the British Pharmaceutical Industry), and AERIS (Acute Exacerbation and Respiratory Infections in COPD) cohorts. The microbiome was analyzed using COPDMAP and AERIS as a discovery data set and BEAT-COPD as a validation data set.

**Measurements and Main Results:** The airway microbiome in neutrophilic COPD was heterogeneous, with two primary community types differentiated by the predominance of *Haemophilus*. The *Haemophilus*-predominant subgroup had elevated sputum IL-1β and TNFα (tumor necrosis factor α) and was relatively stable over time. The other neutrophilic subgroup with a balanced microbiome profile had elevated sputum and serum IL-17A and was temporally dynamic. Patients in this state at stability were susceptible to the greatest microbiome shifts during exacerbations. This subgroup can temporally switch to both neutrophilic *Haemophilus-predominant* and eosinophilic states that were otherwise mutually exclusive. Time-series analysis on the microbiome showed that the temporal trajectories of *Campylobacter* and *Granulicatella* were indicative of intrapatient switches from neutrophilic to eosinophilic inflammation, in track with patient sputum eosinophilia over time. Network analysis revealed distinct host–microbiome interaction patterns among neutrophilic *Haemophilus*-predominant, neutrophilic balanced microbiome, and eosinophilic subgroups.

**Conclusions:** The airway microbiome can stratify neutrophilic COPD into subgroups that justify different therapies. Neutrophilic and eosinophilic COPD are interchangeable in some patients. Monitoring temporal variability of the airway microbiome may track patient inflammatory status over time.

**At a Glance Commentary**

**Scientific Knowledge on the Subject**

Chronic obstructive pulmonary disease (COPD) is heterogeneous. Increasing evidence shows that the airway microbiome is related to COPD clinical phenotypes, severity, and long-term mortality. Understanding the role of the airway microbiome in COPD neutrophilic and eosinophilic inflammatory endotypes may help develop microbiome-based approaches for patient selection for targeted therapeutic intervention. There is a paucity of data examining the dynamic relationships between the airway microbiome and COPD inflammatory endotypes across stability and exacerbations.

**What This Study Adds to the Field**

This study reports an integrated analysis on the airway microbiome in COPD neutrophilic and eosinophilic endotypes using 1,706 sputum samples collected longitudinally from 510 participants with COPD in three UK cohorts in 2008–2015. We showed that two primary types of airway ecology existed in neutrophilic COPD, which differed by the predominance of *Haemophilus*, inflammatory mediators, temporal stability, and interchangeability with eosinophilic inflammation and could therefore justify different therapeutic approaches. There were specific, nondominant microbiome genera associated with eosinophilia. Monitoring temporal variability of these features tracked patient inflammatory status over time, suggesting a potential need for point-of-care diagnosis using sputum microbiome biomarkers. These results highlight the importance of the airway microbiome in the inflammatory endotype–based patient management in COPD.

Chronic obstructive pulmonary disease (COPD) is a heterogeneous disease underpinned by diverse clinical characteristics and pathophysiological mechanisms (1–3), with episodes of exacerbations leading to significant mortality worldwide (4–6). A better understanding of clinical phenotypes and biological endotypes for COPD is crucial for developing precision-medicine strategies that enable patient-tailored treatment according to clinical characteristics coupled with biomarkers of underlying disease mechanisms (7).

Inflammatory patterns observed in individuals with COPD have been referred to as inflammatory endotypes (8). Neutrophilic inflammation is a hallmark of COPD and contributes to key pathological features, including emphysema and mucociliary dysfunction (9). Bacterial infection is associated with neutrophilic inflammation, but its role in driving inflammation is uncertain, given that increased neutrophilia is observed in both colonized and noncolonized patients with COPD (10). Eosinophilic inflammation is also present as a stable endotype in a subgroup of patients with COPD (11) and is associated with less bacterial infection and a favorable response to inhaled corticosteroids (ICSs) (12–14). Individuals with COPD have been broadly classified into neutrophilic, eosinophilic, mixed, and paucigranulocytic inflammation according to differential sputum cell counts (15), yet it is currently unknown whether such a definition sufficiently captures the underlying disease heterogeneity.

Recent studies have demonstrated a diverse airway microbiome associated with COPD severity, exacerbations, clinical phenotypes, and long-term mortality (16–20). The airway microbiota differs between bacteria-associated and eosinophilic exacerbations, with a lower diversity and increased *Proteobacteria* in the former (16, 17), suggesting airway ecology may be correlated with their underlying inflammatory processes. Although the airway microbiome was recently shown to differ between neutrophilic and eosinophilic inflammation in stable asthma (21), the dynamic relationship between the microbiome and inflammatory endotypes across stability and exacerbations in COPD remains unclear and warrants investigation in large, longitudinal cohorts.

We hypothesized that the airway microbiome is differentially associated in neutrophilic and eosinophilic inflammations in COPD and that such associations may be dynamic across stability and exacerbations. We also hypothesized that different airway ecology reflects distinct pathophysiology and may capture additional heterogeneity within the broadly defined inflammatory endotypes and assist in patient stratification. We tested these hypotheses using an integrated analysis on three large-scale longitudinal cohorts established at four clinical sites in the United Kingdom in 2008–2015: BEAT-COPD (Biomarkers to Target Antibiotic and Systemic COPD) (16), COPDMAP (COPD Medical Research Council/Association of the British Pharmaceutical Industry) (22), and AERIS (Acute Exacerbation and Respiratory Infections in COPD) (17). The participants in these cohorts were well characterized and followed at stability and during exacerbations for up to 2 years. A total of 1,706 sputum samples from 510 patients were included in this analysis, which, to our knowledge, represents the largest COPD microbiome analysis to date. Importantly, all samples in COPDMAP and AERIS were processed using the same procedure and platform in the same genomic facility, making them essentially one centralized data resource for collective analysis. In this study, we analyzed the combined COPDMAP and AERIS cohorts as the main discovery data set. Whenever applicable, the results were independently validated in BEAT-COPD, in which the microbiota was characterized using a different platform. The aim of this analysis was to systematically assess the relationship between the airway microbiome and COPD inflammatory endotypes across stability and exacerbations.

## Methods

### Participants and Samples

The procedure for patient recruitment was described in detail previously (16, 17, 22). Participants were recruited if they had a physician diagnosis of COPD with a post-bronchodilator FEV_1_/FVC ratio <70% at screening and no previous asthma diagnosis. BEAT-COPD participants were recruited from the Glenfield Hospital in Leicester, United Kingdom. COPDMAP participants were recruited at Imperial College London, the University of Leicester, and the University Hospital of South Manchester, United Kingdom. AERIS participants were recruited at the University Hospital Southampton, United Kingdom. The stable visits were defined as visits during stable disease, with at least 4 weeks free from a prior exacerbation. Exacerbations were defined according to Anthonisen criteria (23) and/or healthcare use (24). Exacerbation samples were collected before treatment with antibiotics or steroids.

Sputum samples were classified on the basis of established criteria for sputum differential cell counts into four groups (15, 25): neutrophilic (eosinophils < 3%, neutrophils ≥ 61%), eosinophilic (eosinophils ≥ 3%, neutrophils < 61%), mixed-granulocytic (eosinophils ≥ 3%, neutrophils ≥ 61%), and paucigranulocytic (eosinophils < 3%, neutrophils < 61%). A panel of inflammatory mediators was measured in the sputum and serum for a subgroup of patients in COPDMAP (*N* = 157) and BEAT-COPD (*N* = 113) using the Meso Scale Discovery platform (Meso Scale Diagnostics). The measurements were quality controlled as described previously (26).

### Microbiome Sequencing and Analysis

All COPDMAP and AERIS samples were processed in a single, centralized laboratory at GlaxoSmithKline Research and Development) according to the same protocol as described previously (17, 22). The V4 hypervariable region of the 16S rRNA gene was sequenced using Illumina MiSeq with proper reagent controls (*see* online supplement). For BEAT-COPD, the 16S V3–V5 region was sequenced using the 454 Genome Sequencer (454 Life Sciences). All sequencing data were deposited in the National Center for Biotechnology Information Sequence Read Archive (BEAT-COPD: SRP065072, COPDMAP: SRP102480, AERIS: SRP102629). The computer codes for data analyses were provided in the online supplement or deposited in GitHub under https://github.com/wangzlab/AERIS_MAP_BEAT_analysis.

All 16S rRNA gene data sets were processed using a standardized pipeline in QIIME 2.0 (Quantitative Insights Into Microbial Ecology 2.0) (27). The demultiplexed sequencing reads were denoised to generate amplicon sequence variants using DADA2 (Divisive Amplicon Denoising Algorithm 2) (28). Additional denoising parameters were used for 454 data (28). A custom Naive Bayes classifier was trained on Greengenes Database (Second Genome, Inc.) 13_8 99% operational taxonomic units to assign a taxonomy for each data set. The COPDMAP and AERIS samples were rarefied to 29,117 reads. The BEAT-COPD samples were rarefied to 2,207 reads. The sequencing batch information for COPDMAP and AERIS were used to adjust batch effects for microbiome data using Combat (29), according to the method Gibbons and colleagues (30).

### Statistical Analyses

The detailed procedure for statistical analyses is provided in the online supplement. Microbiome community types were identified using unbiased clustering by Wald linkage on the basis of the Jensen-Shannon divergence index. The optimal number of clusters was determined by using the Silhouette measure of the degree of confidence (31). Cluster memberships were validated using a partition around the medoids, with the optimal number of clusters determined by using the Calinski-Harabasz index (32). Changepoint detection analysis was performed using the pruned exact linear time algorithm (33) in the changepoint package in R (R Foundation for Statistical Computing) (34) to search for temporal change points on the relative abundance of microbiome genera. We calculated cross-covariance scores between the relative abundances of microbiome genera and sputum neutrophilic and eosinophilic percentages for all longitudinal visits of each patient using the ccf function in R (35). Patients with at least five visits were included. A microbiome cooccurrence network was established using SparCC (Sparse Correlations for Compositional data) (36) and visualized using Gephi (37). The correlation between microbiome genera and mediators was assessed first by residualization using a general linear mixed model to adjust for demographic covariates and then by hierarchical all-against-all association testing using HAllA (Hierarchical All-against-All association testing) (38).

## Results

### Participant and Sample Characteristics

A total of 1,366 sputum samples were collected from 423 patients with COPD in the COPDMAP and AERIS cohorts, spanning between 1 and 13 visits over up to 2 years (time-span range, 4–658 d; mean, 301.5 d) at clinical stability (*N* = 920) and during exacerbations (*N* = 446) in London (*N* = 300), Leicester (*N* = 303), Manchester (*N* = 180), and Southampton (*N* = 583) in the United Kingdom (*see* Figure E1 in the online supplement and Table 1). As a validation data set, 340 samples from the BEAT-COPD cohort from 87 participants at a stable state (*N* = 203) and during exacerbations (*N* = 137) were included (1–9 visits per participant, time-span range, 5–881 d; mean, 238.4 d; Figure E1 and Table 1).

**Figure 1.**
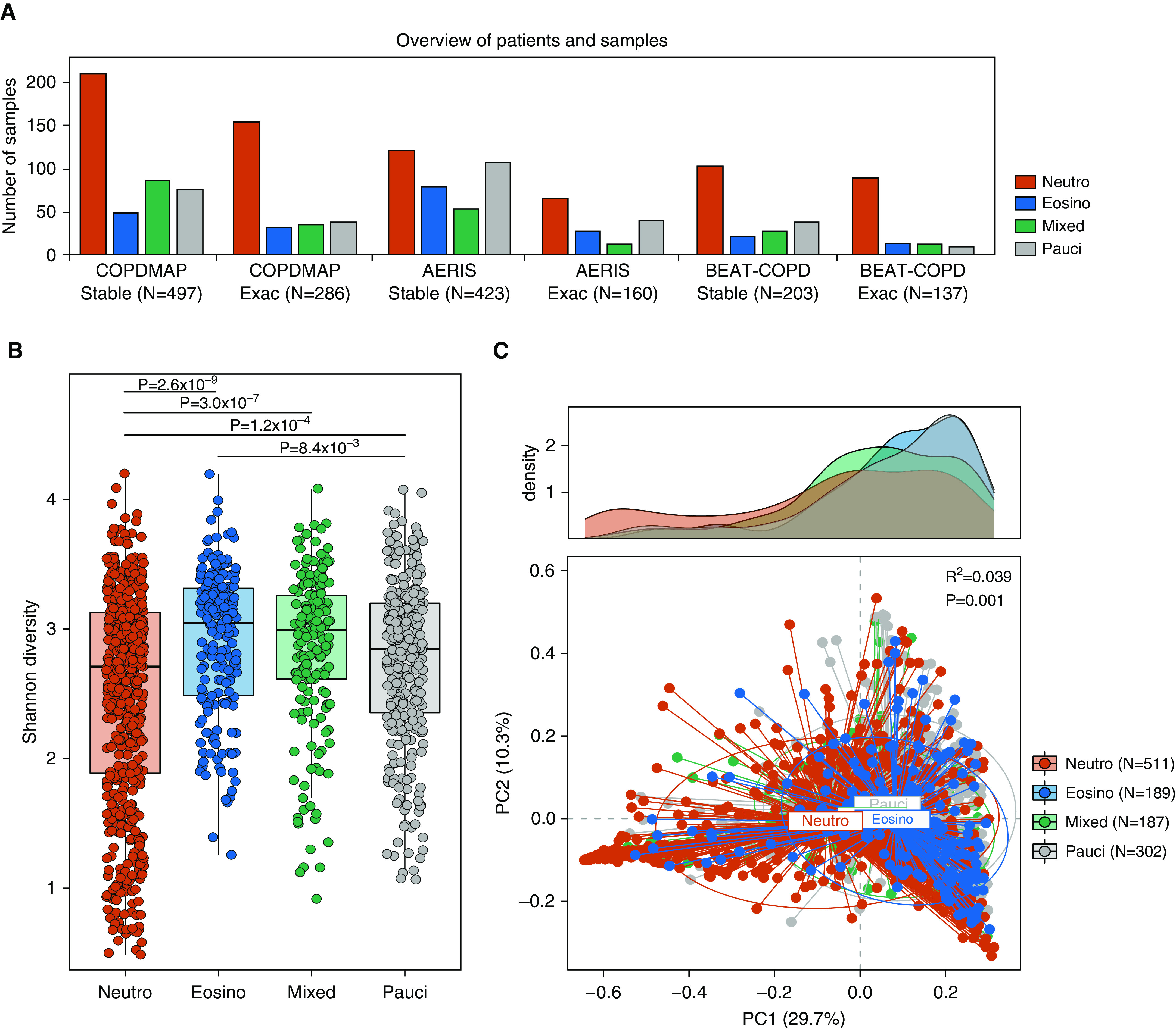
The airway microbiome in neutrophilic (Neutro) and eosinophilic (Eosino) chronic obstructive pulmonary disease (COPD). (*A*) The number of sputum samples in Neutro, Eosino, mixed, and paucigranulocytic (Pauci) subgroups across stability and exacerbations in the COPDMAP (COPD Medical Research Council/Association of the British Pharmaceutical Industry), AERIS (Acute Exacerbation and Respiratory Infections in COPD), and BEAT-COPD (Biomarkers to Target Antibiotic and Systemic COPD) cohorts. (*B*) Shannon diversity of the airway microbiome in sputum samples in the Neutro, Eosino, mixed, and Pauci subgroups. A significantly reduced α diversity was observed for the Neutro subgroup compared with the other groups. (*C*) Principal coordinate (PC) analysis based on Bray-Curtis dissimilarity for samples in four inflammatory subgroups. The microbiome significantly differed across the four groups (permutational ANOVA, *R^2^* = 0.039, *P* = 0.001). The density plot showed a more diverse PC1 distribution for the Neutro subgroup than for the other groups.

**Table 1.** Demographic and Clinical Features of Participants in the COPDMAP, AERIS, and BEAT-COPD Cohorts

Features	COPDMAP			AERIS	BEAT-COPD
	Leicester (*N* = *303*)	London (*N* = *300*)	Manchester (*N* = *180*)	Southampton (*N* = *583*)	Leicester (*N* = *340*)
Number of patients	100	128	94	101	87
Number of visits per patients*	3.0 (1–9)	2.3 (1–8)	1.9 (1–5)	5.8 (1–13)	3.9 (2–9)
Number of exacerbation visits per patients*	1.6 (1–5)	2.0 (1–6)	1.2 (1–3)	2.1 (1–6)	1.6 (1–4)
Age^†^^‡^	68.7 ± 7.6	71.1 ± 8.6	66.3 ± 6.5	67.1 ± 8.4	67.7 ± 9.0
Sex, F^‡^^§^	24 (24.0)	41 (32.0)	26 (27.7)	42 (41.6)	22 (25.3)
BMI^‡^	27.8 ± 5.0	26.7 ± 5.7	26.9 ± 5.1	27.6 ± 5.4	26.4 ± 4.6
Current smokers^‡^	74 (74.0)	89 (69.5)	54 (57.4)	40 (39.6)	37 (42.5)
GOLD status, I/II/III/IV^‡^	9/62/33/11	8/51/32/8	15/40/29/10	0/45/40/16	1/35/32/19
FEV_1_, L	1.5 ± 0.5	1.2 ± 0.5	1.6 ± 0.6	1.2 ± 0.6	1.2 ± 0.5
FEV_1_% predicted	49.2 ± 16.3	48.7 ± 21.6	51.0 ± 20.4	46.7 ± 25.3	44.9 ± 18.7
FVC, L	2.7 ± 0.7	3.0 ± 1.0	3.3 ± 0.9	2.9 ± 0.9	2.5 ± 0.8
FEV_1_/FVC ratio	0.5 ± 0.1	0.5 ± 0.2	0.5 ± 0.4	0.4 ± 0.2	0.5 ± 0.1
Questionnaire, CAT or CRQ	21.9 ± 6.8^ǁ^	19.9 ± 7.8^ǁ^	21.1 ± 8.0^ǁ^	16.6 ± 10.0^ǁ^	14.9 ± 5.0^¶^
Sputum neutrophils, %	69.5 ± 24.9	66.8 ± 14.0	72.0 ± 16.0	50.0 ± 36.7	73.0 ± 22.6
Sputum eosinophils, %	2.2 ± 4.0	2.7 ± 4.4	3.1 ± 3.3	3.4 ± 7.7	3.6 ± 7.8
Sputum lymphocytes, %	0.3 ± 0.6	0.7 ± 0.9	0.2 ± 0.3	0.3 ± 0.8	0.5 ± 0.8
Sputum macrophages, %	17.7 ± 18.7	24.4 ± 19.2	13.4 ± 15.0	19.5 ± 18.9	19.8 ± 18.2
Sputum epithelial cells, %	4.9 ± 6.6	1.3 ± 2.8	3.6 ± 4.4	3.9 ± 5.5	3.1 ± 6.1
Blood neutrophils, 10^9^ cells/L	5.3 ± 2.1	5.9 ± 2.5	4.8 ± 1.7	4.8 ± 1.7	6.2 ± 2.5
Blood eosinophils, 10^9^ cells/L	0.2 ± 0.2	0.2 ± 0.2	0.2 ± 0.2	0.2 ± 0.2	0.3 ± 0.2
Blood lymphocytes, 10^9^ cells/L	1.9 ± 0.7	1.8 ± 0.7	1.7 ± 0.6	1.8 ± 0.8	2.1 ± 1.0
Blood monocytes, 10^9^ cells/L	0.5 ± 0.2	0.8 ± 0.3	0.6 ± 0.2	0.7 ± 0.3	0.6 ± 0.2
Blood basophils, 10^9^ cells/L)	0.1 ± 0.0	0.0 ± 0.0	0.0 ± 0.0	0.0 ± 0.0	0.0 ± 0.0
Community type, Ba/Hi/Mc/Sp, %**	75/14/7/4	74/13/6/7	72/17/7/4	63/21/7/9	59/17/9/15
Inflammatory group, Neu/Eos/Mix/Pau, %**	57/10/13/20	51/12/20/17	49/18/23/10	34/20/12/34	61/11/13/15
Bacterial infection during exacerbations^††^	37 (45.1)	54 (32.0)	15 (51.7)	79 (49.4)	51 (45.5)
Viral infection during exacerbations^††^	14 (17.0)	32 (18.9)	5 (17.2)	27 (16.8)	30 (26.8)
ICS usage at enrolment^‡‡^	90 (90.0)	102 (80.0)	74 (78.7)	94 (93.1)	67 (77.0)
Macrolide usage at stability^‡‡^	4 (4.0)	2 (1.6)	1 (1.1)	6 (5.9)	0 (0.0)
Number of spontaneous sputum samples	291 (96.0)	296 (98.7)	169 (93.9)	443 (76.0)	329 (96.8)

*Definition of abbreviations*: AERIS = Acute Exacerbation and Respiratory Infections in COPD; Ba = balanced; BEAT-COPD = Biomarkers to Target Antibiotic and Systemic COPD; BMI = body mass index; CAT = COPD Assessment Test; COPD = chronic obstructive pulmonary disease; COPDMAP = COPD Medical Research Council/Association of the British Pharmaceutical Industry; CRQ = Chronic Respiratory Disease Questionnaire; Eos = eosinophilic; GOLD = Global Initiative for Chronic Obstructive Lung Disease; Hi = *Haemophilus*-predominant; ICS = inhaled corticosteroid; Mc = *Moraxella*-predominant; Mix = mixed-granulocytic; Neu = neutrophilic; Pau = paucigranulocytic; Sp = *Streptococcus*-predominant.

^*^ Presented as mean (range).

^†^ Continuous data are presented as the mean ± SD unless otherwise stated.

^‡^ Patient demographic data at baseline.

^§^ Categorical data are presented as the number (proportion) unless otherwise stated.

^ǁ^ CAT score.

^¶^ CRQ score.

^**^ Percentage proportion of samples in each subgroup.

^††^ Bacterial and viral infections at exacerbations were defined according to Bafadhel and colleagues (26).

^‡‡^ Number of patients.

Chronic use of macrolides was reported for antiinflammatory purposes for 16 stable visits from 13 participants in COPDMAP and AERIS. No macrolide use was reported for BEAT-COPD participants. No significant associations were found between the microbiota and chronic macrolide usage (Figure E2). No significant differences in the microbiota were observed between induced and spontaneous sputum samples at a stable state (Figure E3).

**Figure 2.**
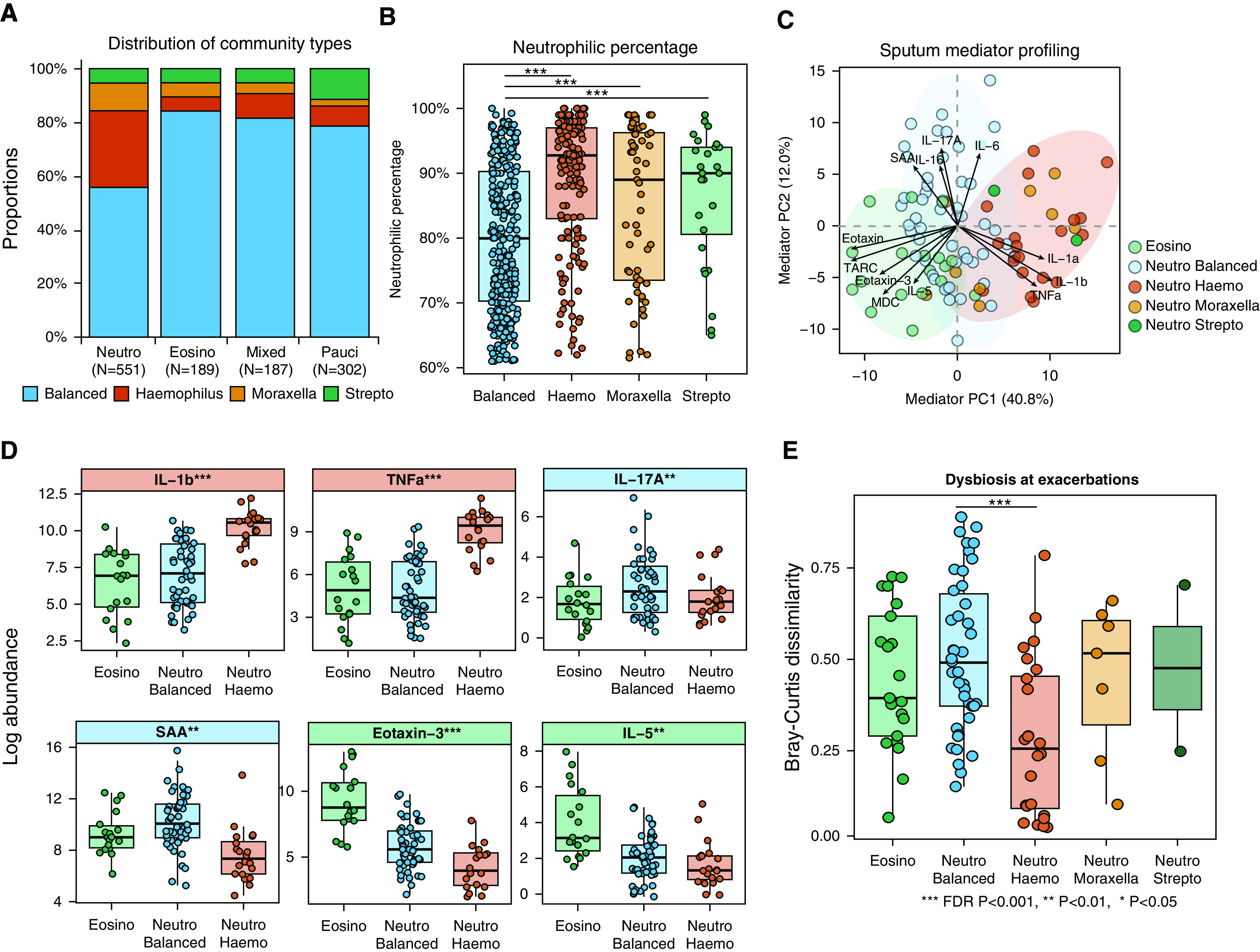
The microbiome in neutrophilic (Neutro) chronic obstructive pulmonary disease (COPD) was heterogeneous. (*A*) The distribution of four community types across Neutro, eosinophilic (Eosino), mixed, and paucigranulocytic (Pauci) subgroups in the COPDMAP (COPD Medical Research Council/Association of the British Pharmaceutical Industry) and AERIS (Acute Exacerbation and Respiratory Infections in COPD) cohorts. (*B*) The sputum Neutro percentage in Neutro samples across four community types in the COPDMAP and AERIS cohorts. There was a significantly decreased Neutro percentage in the Neutro balanced subgroup compared with the other subgroups. (*C*) Principal component analysis on sputum mediators for the Neutro and Eosino subgroups in COPDMAP. Neutro samples were further colored according to their microbiome community types. The Neutro *Haemophilus *(Haemo), Neutro balanced, and Eosino subgroups were clustered separately. (*D*) Box-and-whisker plots showing the sputum mediators most elevated in the Neutro Haemo, Neutro balanced, and Eosino subgroups in COPDMAP. (*E*) The greatest Bray-Curtis dissimilarity for paired stability–exacerbation samples in the Neutro balanced subgroup compared with the other subgroups in the COPDMAP and AERIS cohorts, indicating the greatest microbiome shifts during exacerbations in this subgroup. *False discovery rate (FDR) *P* < 0.05, **FDR *P* < 0.01, and ***FDR *P* < 0.001. PC = principal component; SAA = serum amyloid A; Strepto = *Streptococcus*.

**Figure 3.**
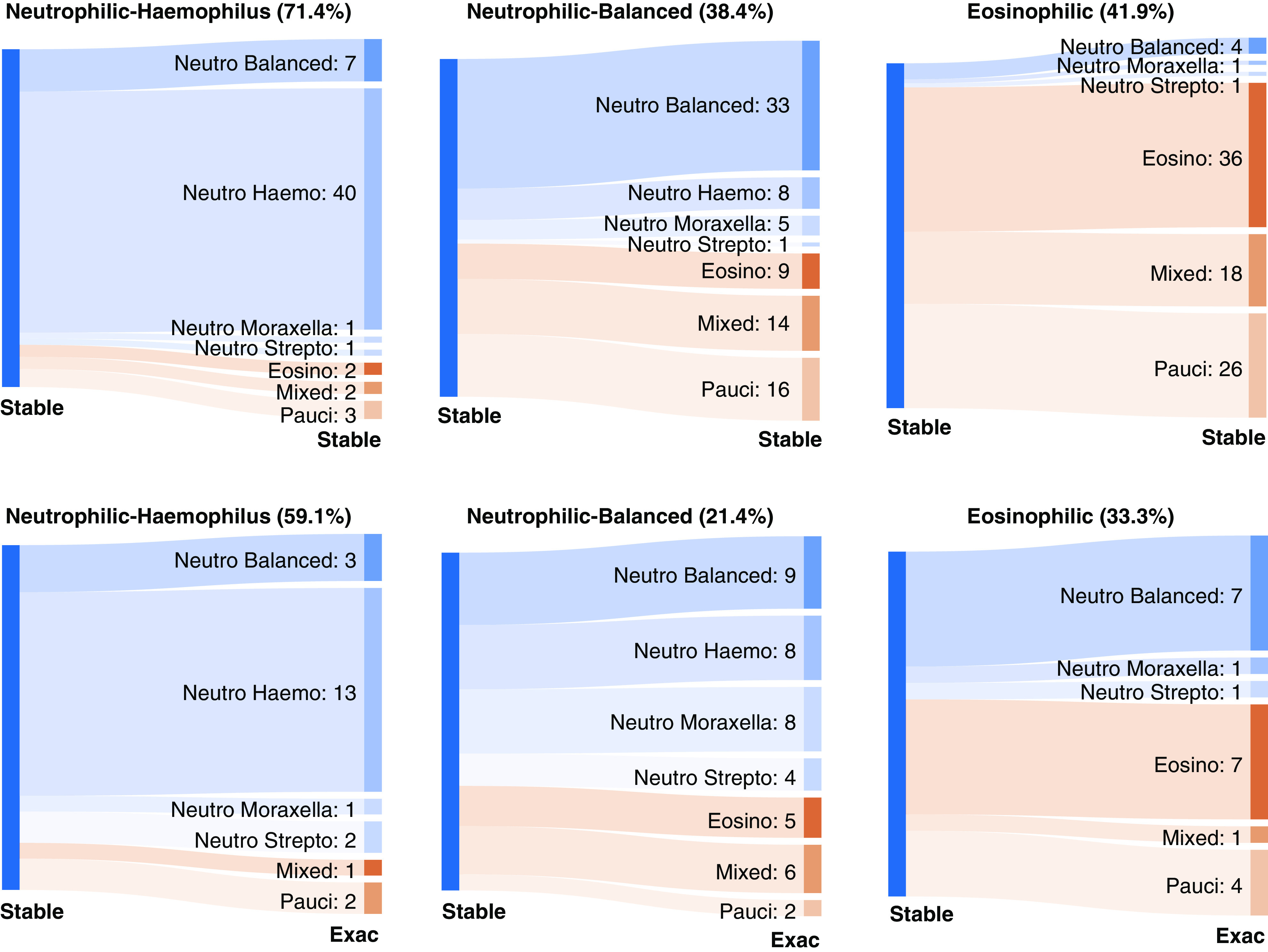
The neutrophilic (Neutro) balanced microbiome subgroup was temporally dynamic. Sankey diagrams showing the temporal transition pattern for Neutro *Haemophilus *(Haemo), Neutro balanced, and eosinophilic (Eosino) states within stable disease (Stable–Stable) and between stability and exacerbation (Exac) (Stable–Exac) in the COPDMAP (COPD Medical Research Council/Association of the British Pharmaceutical Industry) and AERIS (Acute Exacerbations and Respiratory Infections in COPD) cohorts. The width of the band is proportional to the number of samples transitioned to the corresponding subgroup. The proportion of samples that remained in the same state is indicated in parentheses. The Neutro balanced subgroup was interchangeable with both Neutro *Haemo* and Eosino subgroups that were otherwise mostly mutually exclusive. Pauci = paucigranulocytic; Strepto = *Streptococcus*.

### Neutrophilic COPD Was Heterogeneous with Differential Airway Ecology

We classified 1,366 samples in the discovery data set into neutrophilic (*N* = 551), eosinophilic (*N* = 189), mixed (*N* = 187), and paucigranulocytic (*N* = 302) subgroups. Of these, 137 samples could not be assigned to a subgroup because of missing data. The 340 samples in BEAT-COPD were also classified accordingly. The distribution of sputum differential cell counts was generally comparable across cohorts and sites (Figure E4), suggesting a degree of consistency between the independent assessments. The distribution of each subgroup was comparable across cohorts and sites, except for a relatively higher proportion of paucigranulocytic samples in AERIS (Figures 1A and E5A).

**Figure 4.**
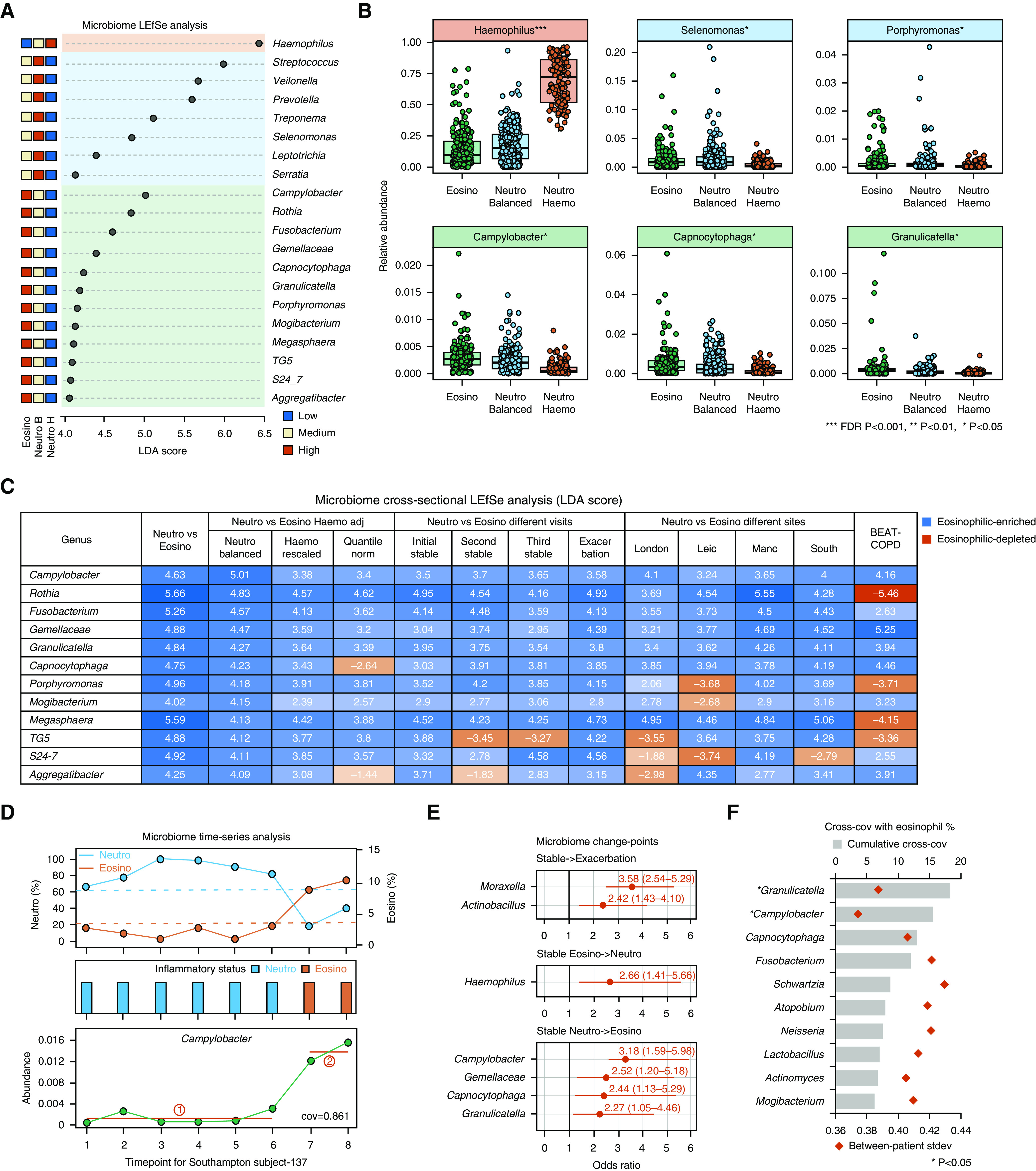
Specific nondominant microbiome genera were associated with eosinophilia. (*A*) Linear discriminant analysis (LDA) effect size (LEfSe) analysis showing microbiome genera specifically enriched in neutrophilic (Neutro) *Haemophilus *(Haemo), Neutro balanced (B), and eosinophilic (Eosino) subgroups (LDA > 4.0, false discovery rate [FDR] *P* < 0.05). The average relative abundances ranked from high to low are shown for each genus across the three subgroups in the COPDMAP (Chronic Obstructive Pulmonary Disease [COPD] Medical Research Council/Association of the British Pharmaceutical Industry) and AERIS (Acute Exacerbation and Respiratory Infections in COPD) cohorts. (*B*) Box-and-whisker plots showing microbiome genera most enriched in each subgroup (LDA, FDR *P* < 0.05). (*C*) LEfSe analysis showing enrichment (blue) or depletion (red) of the 12 genera in the Eosino versus Neutro group in multiple analyses by *1*) comparing the Eosino and Neutro B subgroups and using two additional approaches to control for Haemo overgrowth in the Neutro group, by rescaling relative abundances with Haemo abundance downscaled to its average across samples according to the method used by Taylor and colleagues (21) (Haemo rescaled), and by using a Quantile norm approach to rescale relative abundances to their within-sample percentile ranks; *2*) subanalyzing within the initial, second, and third stable visits and during exacerbations; *3*) subanalyzing within each of the four sites; and *4*) using BEAT-COPD (Biomarkers to Target Antibiotic and Systemic COPD) data. The LDA score for each specific comparison is indicated in the corresponding cell in the table. (*D*) An example illustrating the time-series analysis on longitudinal microbiome data. Shown are the changes in relative abundances of *Campylobacter* compared with the changes in Neutro and Eosino status across visits for one patient (South subject-137). The break in between the red lines indicates significant changes in the relative abundance of *Campylobacter* identified by the changepoint-detection algorithm, which coincided with the switch from the Neutro to the Eosino state. The changes in abundance of *Campylobacter* were also in concert with sputum Eosino percentages over time, with a cross-covariance (cross-cov) score of 0.861. (*E*) The microbiome genera whose change points were associated with exacerbation events and with switches between Neutro and Eosino inflammation within stable disease. The odds ratio and 95% confidence interval (95% CI) are shown. Only significant genera with lower limit of the 95% CI greater than 1.0 are shown. (*F*) The top 10 genera with greatest cumulative cross-cov scores with sputum Eosino percentages. The cumulative cross-cov score and interpatient stdev of the scores were shown for each genus. The genera were highlighted in asterisks if their cross-cov scores were significantly higher than the null distributions derived from permutation test. *FDR *P* < 0.05. **FDR *P* < 0.01, and ***FDR *P* < 0.001. adj = adjusted; H = Haemo-predominant; Leic = Leicester; Manc = Manchester; Quantile norm = quantile normalization; South = Southampton; stdev = SD.

Conducting α-diversity analysis on the discovery data set revealed an overall significantly decreased Shannon index in the neutrophilic group compared with the other three groups (Wilcoxon *P* ≤ 8.4 × 10^−3^, Figure 1B). However, there was a broad range of Shannon index values in the neutrophilic group, with a subset of samples without a notable reduction in α diversity. This was also manifested by a significantly greater deviation from the centroid in the neutrophilic group compared with the other groups in principal coordinate analysis of microbiome β-diversity (Bray-Curtis dissimilarity, Figures 1C and E5B). A similar pattern was observed in BEAT-COPD (Figures E6A and E6B). A similar pattern was observed when analyzing stable and exacerbation samples separately (Figure E7). These results suggested that there were heterogeneous patterns of the microbiome within the neutrophilic group.

To dissect the heterogeneity of the microbiome in the neutrophilic group, we performed an unsupervised clustering of microbiome profiles into community types. Hierarchical clustering on all 1,366 microbiome profiles revealed an optimum of four community classes. The main community type was composed of a “balanced” microbial composition, whereas the other three types had a “biased” composition dominated by *Haemophilus*, *Moraxella*, and *Streptococcus*, respectively (Figure E8A). These three community types were overall in agreement with *H. influenzae*, *M. catarrhalis*, and *S. pneumoniae* colonization on the basis of quantitative PCR and culture results, except for a relatively low culture positivity for *S. pneumoniae* (Figure E9). Similar community clusters were also identified using the partition-around-the-medoids clustering method and in BEAT-COPD (Figures E8A–E8C), suggesting robustness of the community types. The four community types persisted when analyzing stable or exacerbation samples alone (Figure E10A).

The relative abundance of *Haemophilus* and the quantitative-PCR load of *H. influenzae* had areas under the curve (AUCs) of 0.949 and 0.908 in distinguishing the *Haemophilus*-predominant subgroup from the remaining samples in the discovery data set (AUCs of 0.999 and 0.912 in the validation data set). The *Haemophilus* relative abundance of 0.41 and log_10_ *H. influenzae* load of 7.2 (copies/ml) reached the best discriminatory power for both data sets. In comparison, *Veillonella* and *Prevotella* were most significantly enriched in the balanced microbiome subgroup in both data sets. An added-up relative abundance of *Veillonella* and *Prevotella* of 0.26 reached the highest power in segregating the balanced microbiome subgroup in the discovery data set. In the validation data set, the optimal *Veillonella*+*Prevotella* relative abundance for the subgroup was 0.17.

Next, we compared microbiome communities across inflammatory endotypes (Figures 2A–2C). The majority of samples in the eosinophilic, mixed, and paucigranulocytic groups had a balanced community type (77.1–83.5%, Figure 2A). In comparison, 56.8% of samples in the neutrophilic group had a balanced community type, whereas 27.8% of the samples were *Haemophilus* predominant (Figure 2A). The *Moraxella-* and *Streptococcus*-predominant communities constituted 13.8% of all samples. The distribution of community classes across inflammatory endotypes was overall consistent between stable and exacerbation samples, with a relatively lower representation of the “balanced” community for exacerbations (Figure E10B). We therefore concluded that there were two major types of airway ecology for neutrophilic COPD, which were differentiated by the predominance of *Haemophilus*. They were considered as two neutrophilic subgroups in subsequent analyses (named as the neutrophilic *Haemophilus* and neutrophilic balanced subgroups).

### Neutrophilic COPD with Balanced Microbiome Had Elevated IL-17A

The two neutrophilic subgroups were overall comparable in demographic features and severity in terms of spirometric findings and symptom scores (Tables 2 and E1). There was a significantly lower neutrophilic percentage and a higher eosinophilic percentage in the neutrophilic balanced subgroup compared with the neutrophilic *Haemophilus* subgroup (Figure 2B; Wilcoxon *P* < 0.001). However, the neutrophilic percentage alone cannot differentiate between the two subgroups (AUC, 0.620). In COPDMAP, 37 sputum and serum inflammatory mediators were measured for 157 samples from 80 participants. Principal component analysis on the sputum mediators revealed distinct clusters for the neutrophilic balanced, neutrophilic *Haemophilus*, and eosinophilic subgroups (Figure 2C). In the eosinophilic subgroup, eotaxin-3, TARC (thymus- and activation-regulated chemokine), and IL-5 were elevated, indicating a T-helper cell type 2 (Th2) signature. In the neutrophilic *Haemophilus* subgroup, IL-1α, IL-1β, and TNFα were elevated (false discovery rate [FDR] *P* < 0.001). In the neutrophilic balanced subgroup, IL-17A was most elevated (FDR *P* = 0.04), followed by SAA (serum amyloid A), Flt-1 (fms-related receptor tyrosine kinase 1), and IL-16 (Figures 2D and E11 and Table 2). A similar clustering pattern was also observed in BEAT-COPD, with elevated IL-17A in the neutrophilic balanced subgroup (Figure E12). A similar mediator clustering pattern was further observed for stable or exacerbation samples alone (Figure E13). Among serum mediators, elevated IL-17A and GM-CSF (granulocyte-macrophage colony-stimulating factor) were observed in the neutrophilic balanced subgroup, and IL-5 and eotaxin-3 were higher in the eosinophilic subgroup, both in COPDMAP and BEAT-COPD (Figure E14 and Table E2).

**Table 2.** Comparisons between Patient Demographic and Clinical Features and between Sputum Mediators among the NH, NB, and E Subgroups in the COPDMAP and AERIS Cohorts

Features	NH (*N* = *153*)	NB (*N* = *313*)	E (*N* = *189*)	*P* Value (*NH vs. NB*)	*P* Value (*NH vs. E*)	*P* Value (*NB vs. E*)
Number of patients*	34	95	43	NA	NA	NA
Number of exacerbation visits	60 (39.2)	110 (35.1)	62 (32.6)	0.39	0.22	0.59
Age^†^^‡^	69.3 ± 9.0	68.5 ± 7.6	66.9 ± 7.8	0.67	0.21	0.24
Sex, F^‡^^§^	8 (23.5)	29 (30.5)	11 (25.6)	0.44	0.89	0.55
BMI^‡^	26.8 ± 5.8	27.0 ± 6.1	26.9 ± 4.4	0.81	0.91	0.89
Current smokers^‡^	24 (69.7)	62 (65.3)	26 (60.5)	0.57	0.35	0.59
GOLD status, I/II/III/IV^‡^	4/15/12/3	16/39/26/14	10/15/12/6	0.64	0.47	0.81
ICS usage at enrolment^‡^	29 (85.2)	82 (86.3)	37 (86.0)	0.88	0.93	0.97
Macrolide use at stability^‡^	3 (8.8)	5 (5.3)	1 (2.3)	0.46	0.20	0.43
FEV_1_, L	1.2 ± 0.7	1.3 ± 0.5	1.4 ± 0.6	0.47	0.17	0.29
FEV_1_% predicted	48.0 ± 10.7	49.1 ± 11.3	49.6 ± 10.6	0.67	0.17	0.63
FVC, L	2.6 ± 0.9	2.7 ± 0.9	2.8 ± 0.9	0.14	0.07	0.52
FEV_1_/FVC ratio	0.4 ± 0.2	0.5 ± 0.2	0.5 ± 0.2	0.27	0.19	0.31
CAT score	22.3 ± 5.7	21.1 ± 7.3	20.9 ± 8.5	0.19	0.09	0.79
Sputum neutrophils, %	88.8 ± 10.3	80.5 ± 11.3	38.7 ± 21.1	<0.001	<0.001	<0.001
Sputum eosinophils, %	0.6 ± 0.7	0.9 ± 0.9	10.1 ± 11.7	<0.001	<0.001	<0.001
Sputum lymphocytes, %	0.3 ± 1.0	0.5 ± 1.7	0.4 ± 0.7	0.021	0.32	0.08
Sputum macrophages, %	6.3 ± 5.1	10.7 ± 8.2	30.1 ± 18.4	<0.001	<0.001	<0.001
Sputum epithelial cells, %	2.2 ± 3.6	3.6 ± 5.4	6.0 ± 5.9	0.011	<0.001	<0.001
Blood neutrophils, 10^9^ cells/L	6.0 ± 2.6	5.8 ± 2.6	4.3 ± 1.4	0.65	<0.001	<0.001
Blood eosinophils, 10^9^ cells/L	0.2 ± 0.1	0.2 ± 0.1	0.4 ± 0.2	0.86	<0.001	<0.001
Blood lymphocytes, 10^9^ cells/L	0.8 ± 0.7	1.0 ± 0.6	1.2 ± 0.9	0.05	<0.001	0.02
Blood monocytes, 10^9^ cells/L	1.6 ± 0.7	1.4 ± 0.8	1.4 ± 0.8	0.13	0.10	0.10
Blood basophils, 10^9^ cells/L	0.0 ± 0.0	0.0 ± 0.0	0.0 ± 0.0	0.15	0.36	0.11
Bacterial infection during exacerbations^ǁ^	50 (83.3)	31 (28.1)	11 (18.0)	<0.001	<0.001	<0.001
Viral infection during exacerbations^ǁ^	19 (31.7)	23 (20.9)	19 (31.1)	0.12	0.95	0.14
Bfgf^¶^	1.7 ± 0.8	1.3 ± 1.0	1.7 ± 0.8	0.11	0.82	0.11
CRP	12.2 ± 3.3	10.7 ± 3.4	11.6 ± 2.5	0.15	0.73	0.32
Eotaxin	4.2 ± 2.0	5.4 ± 1.3	7.1 ± 1.5	0.02	<0.001	<0.001
Eotaxin-3	5.0 ± 2.0	5.6 ± 1.9	8.7 ± 2.8	0.25	<0.001	<0.001
Flt-1	4.7 ± 1.2	5.8 ± 1.6	6.0 ± 1.7	0.005	0.001	0.24
GM-CSF	1.0 ± 0.7	0.8 ± 0.7	1.0 ± 0.9	0.33	0.99	0.29
ICAM-1	11.2 ± 1.8	12.3 ± 2.1	13.2 ± 1.8	0.01	<0.001	0.07
IFN	5.3 ± 1.7	4.1 ± 2.1	4.0 ± 1.9	0.008	0.008	0.88
IL-10	5.6 ± 1.9	3.0 ± 1.8	3.0 ± 1.7	<0.001	<0.001	0.96
IL-12 (p70)	2.1 ± 0.6	1.7 ± 0.7	1.7 ± 1.0	0.005	0.05	0.84
IL-12/23 (p40)	3.6 ± 1.0	3.5 ± 1.6	3.4 ± 1.3	0.59	0.39	0.91
IL-13	4.3 ± 0.7	4.2 ± 0.6	4.2 ± 0.6	0.279	0.003	0.99
IL-15	2.8 ± 0.9	2.0 ± 0.7	2.3 ± 0.6	0.001	0.09	0.06
IL-16	5.5 ± 1.9	7.4 ± 2.0	8.4 ± 1.9	0.006	<0.001	0.03
IL-17A	1.8 ± 1.1	2.4 ± 1.6	1.7 ± 1.2	0.004	0.98	0.46
IL-1α	8.1 ± 2.0	6.4 ± 2.2	6.2 ± 1.9	0.004	0.01	0.87
IL-1β	9.6 ± 2.2	7.2 ± 2.2	6.8 ± 2.2	<0.001	<0.001	0.59
IL-2	2.7 ± 1.0	2.1 ± 1.0	2.3 ± 0.8	0.007	0.10	0.34
IL-4	1.0 ± 0.5	0.6 ± 0.4	0.7 ± 0.3	<0.001	0.07	0.13
IL-5	1.9 ± 1.3	2.0 ± 1.3	3.7 ± 2.2	0.51	0.003	0.003
IL-6	6.3 ± 1.5	5.8 ± 1.7	5.6 ± 1.6	0.27	0.42	0.86
IL-7	3.1 ± 1.1	3.6 ± 0.9	4.1 ± 1.1	0.01	0.02	0.25
IL-8	15.7 ± 1.1	14.6 ± 1.4	13.8 ± 1.1	0.001	0.004	0.94
MCP-1	7.9 ± 1.8	8.6 ± 1.9	9.1 ± 1.5	0.16	0.05	0.54
MCP-4	3.6 ± 1.0	3.8 ± 1.2	4.8±1.2	0.99	0.004	0.002
MDC	7.5 ± 1.8	8.1 ± 1.8	9.4 ± 1.7	0.32	0.001	0.004
MIP-1a	8.0 ± 3.0	6.9 ± 2.9	7.8 ± 2.3	0.09	0.54	0.26
MIP-1b	9.1 ± 2.4	8.5 ± 2.3	8.8 ± 3.1	0.17	0.91	0.22
PIGF	4.0 ± 1.0	4.5 ± 1.6	5.0 ± 1.1	0.15	0.005	0.08
SAA	7.6 ± 2.2	10.6 ± 2.2	8.7 ± 1.7	0.005	0.005	0.81
TARC	3.3 ± 2.3	4.5 ± 1.7	6.8 ± 2.0	0.03	<0.001	<0.001
Tie-2	8.0 ± 1.0	6.8 ± 2.3	7.9 ± 2.4	0.08	0.60	0.06
TNFα	8.5 ± 2.2	5.3 ± 2.4	4.9 ± 2.2	<0.001	<0.001	0.55
TNFβ	0.6 ± 0.4	0.3 ± 0.4	0.2 ± 0.2	<0.001	0.004	0.71
VCAM-1	6.8 ± 1.6	7.9 ± 2.0	9.5 ± 2.3	0.04	<0.001	0.008
VEGF-C	6.7 ± 1.2	7.1 ± 1.5	7.2 ± 1.2	0.47	0.47	0.89
VEGF-D	5.5 ± 1.2	5.8 ± 1.7	6.0 ± 1.0	0.34	0.19	0.80

*Definition of abbreviations*: AERIS = Acute Exacerbation and Respiratory Infections in COPD; BMI = body mass index; CAT = COPD Assessment Test; COPD = chronic obstructive pulmonary disease; COPDMAP = COPD Medical Research Council/Association of the British Pharmaceutical Industry; E = eosinophilic; GOLD = Global Initiative for Chronic Obstructive Lung Disease; ICS = inhaled corticosteroid; NB = neutrophilic balanced; NH = neutrophilic *Haemophilus*; SAA = serum amyloid A; TNF = tumor necrosis factor.

^*^ Number of patients whose initial baseline samples belong to the subgroup.

^†^ Continuous data are presented as the mean ± SD.

^‡^ Patient demographic data at baseline or at stability.

^§^ Categorical data are presented as the number (proportion).

^ǁ^ Bacterial and viral infections during exacerbations were defined according to Bafadhel and colleagues (26).

^¶^ The sputum-mediator measurements were only available for COPDMAP.

### Neutrophilic COPD with Balanced Microbiome Was Temporally Dynamic

We assessed within-patient stability for the neutrophilic and eosinophilic endotypes over time by analyzing paired samples collected sequentially from the same patients. Samples were excluded if they did not have a paired sample or if their inflammatory states were not putatively neutrophilic or eosinophilic (i.e., mixed or paucigranulocytic). Within stable disease, 71.4% of neutrophilic *Haemophilus* states were followed by the same state in the next visit, suggesting relative stability (Figure 3). In comparison, 38.4% and 41.8% of neutrophilic balanced and eosinophilic states were succeeded by the same state during stability. In addition, 59.1%, 21.4%, and 33.3% of neutrophilic *Haemophilus,* neutrophilic balanced, and eosinophilic states at stability were maintained during exacerbations. For 47.6% of neutrophilic COPD cases with a balanced microbiome, their communities switched to those dominated by *Haemophilus*, *Moraxella*, or *Streptococcus* during exacerbations. Consistent with this finding, the stable samples in the neutrophilic balanced group had the greatest microbiome shifts during exacerbations compared with other groups, as measured by Bray-Curtis dissimilarity for paired stability–exacerbation samples, both in the discovery data set and in the validation data set (Figures 2E and E12E). These results suggested that the neutrophilic balanced subgroup was dynamic, and patients in this state at stability were most susceptible to airway microbiome shifts during exacerbations. We noted that the neutrophilic *Haemophilus* and eosinophilic states were rarely transited to each other at stability or during exacerbations, indicating their mutually exclusive nature (Figure 3), whereas the neutrophilic balanced state was interchangeable to both neutrophilic Haemophilus and eosinophilic states. Similar transition pattern was observed in BEAT-COPD (Figure E15). Seasonal changes and baseline ICS usage had nonsignificant positive associations with intrapatient switches between neutrophilic and eosinophilic states (stable–stable odds ratios [ORs], 1.60 and 2.22; 95% confidence intervals [95% CIs], 0.63–4.09 and 0.47–10.36; *P* = 0.32 and 0.29; stable-exacerbation ORs, 1.55 and 1.30; 95% CIs, 0.48–5.07 and 0.24–6.78; *P* = 0.46 and 0.78).

### Eosinophilic COPD Was Enriched with Specific Nondominant Genera

Thirty-one genera had a significantly higher relative abundance in the eosinophilic group versus the neutrophilic group (linear discriminant analysis [LDA] effect size, LDA > 4.0; FDR *P* < 0.05; Table E2). Twelve of the 31 genera remained significant between the neutrophilic balanced and eosinophilic groups (LDA > 4.0; FDR *P* < 0.05; Figures 4A–4C and Table E3), indicating that their enrichments in eosinophilia over neutrophilia were independent of *Haemophilus* predominance in the latter. All 12 genera remained eosinophilically enriched using two additional data-normalization approaches to control for *Haemophilus* overgrowth (online supplement; Figure 4C). Furthermore, all 12 genera except for TG5 and *Aggregatibacter* remained eosinophilically enriched when analyzed cross-sectionally within each sequential stable or exacerbation visit. All 12 genera except for S24-7 remained eosinophilically enriched within at least three of four sites. Leicester and London shared a more similar microbiota–eosinophilia correlation pattern than they did with the other sites, which may possibly be related to their relatively smaller proportion of samples in the eosinophilic subgroup (Table 2). We noted that *Mogibacterium*, TG5, and S24-7 had higher abundances in patients with baseline ICS usage; however, these changes were nonsignificant (Figure E16). *Gemellaceae*, *Granulicatella*, *Campylobacter*, *Porphyromonas*, *Capnocytophaga*, and *Rothia* further exhibited significant positive correlations with the sputum eosinophilic percentage in a multivariate linear mixed model for all 1,229 discovery samples (*P* < 0.05), which together explained 35.6% of the variation in sputum eosinophilia (*R*^2^ = 0.356). Patient smoking history was a significant covariate (*P* = 0.018, Table E4). These results suggested that there were nondominant taxa specifically associated with sputum eosinophilia, which were generally robust across sites, visits, and demographics. The enrichments of *Granulicatella*, *Campylobacter*, *Gemellaceae*, and *Capnocytophaga* in the eosinophilic group were validated in BEAT-COPD (Figure E17).

### Microbiome Altered Temporally in Concert with Patient Inflammatory Status

Knowing that neutrophilic and eosinophilic inflammations were interchangeable within patients, we next assessed whether the airway microbiome altered alongside such changes in the patient inflammatory status. We performed a changepoint detection analysis (Figure 4D). As a time-series statistical approach, this analysis divides longitudinal data into segments and identifies points when the distribution of features (i.e., microbiome genera) changes significantly between segments. Ninety-one participants with at least five longitudinal visits were included. Across stability and exacerbations, temporal trajectories of *Moraxella* and *Actinobacillus* were associated with the occurrence of exacerbation events (ORs, 3.58 and 2.42; 95% CIs, 2.54–5.29 and 1.43–4.10; *P* < 0.05, Figure 4E). No genera were associated with switches between neutrophilic and eosinophilic states when viewed across stability and exacerbations, indicating exacerbation had a major impact on temporal variability of the microbiota. When assessing microbiome change points within stability, *Haemophilus* was associated with switches from the eosinophilic to the neutrophilic state (OR, 2.66; 95% CI, 1.41–5.66; *P* = 0.01), whereas *Campylobacter*, *Gemellaceae*, *Capnocytophaga*, and *Granulicatella* were associated with neutrophilic to eosinophilic switches (OR ≥ 2.27, *P* < 0.05, Figure 4E).

To further assess whether the microbiome changed in concert with the extent of neutrophilic and eosinophilic inflammation, we calculated the cross-covariance between temporal measures of the microbiome genera and sputum neutrophilic and eosinophilic percentages for each patient. Given two temporal measures, the cross-covariance estimates the covariation of one measure against the other at pairs of time points (Figure 4D). *Granulicatella* and *Campylobacter* had the highest cumulative cross-covariance with eosinophilia across all participants, which was significantly higher against the null distribution generated by the permutation test (Wilcoxon *P* < 0.05, Figure 4F and Table E5). This was consistent with the markedly lower interpatient variations of their cross-covariance scores and suggested between-patient consistency in their temporal covariation with eosinophilia (Figure 4F). *Moraxella* and *Haemophilus* exhibited the highest cumulative cross-covariance with neutrophilia. However, neither of them reached statistical significance (Table E5).

### Distinct Host–Microbiome Interactions among Inflammatory Subtypes

**Figure 5.**
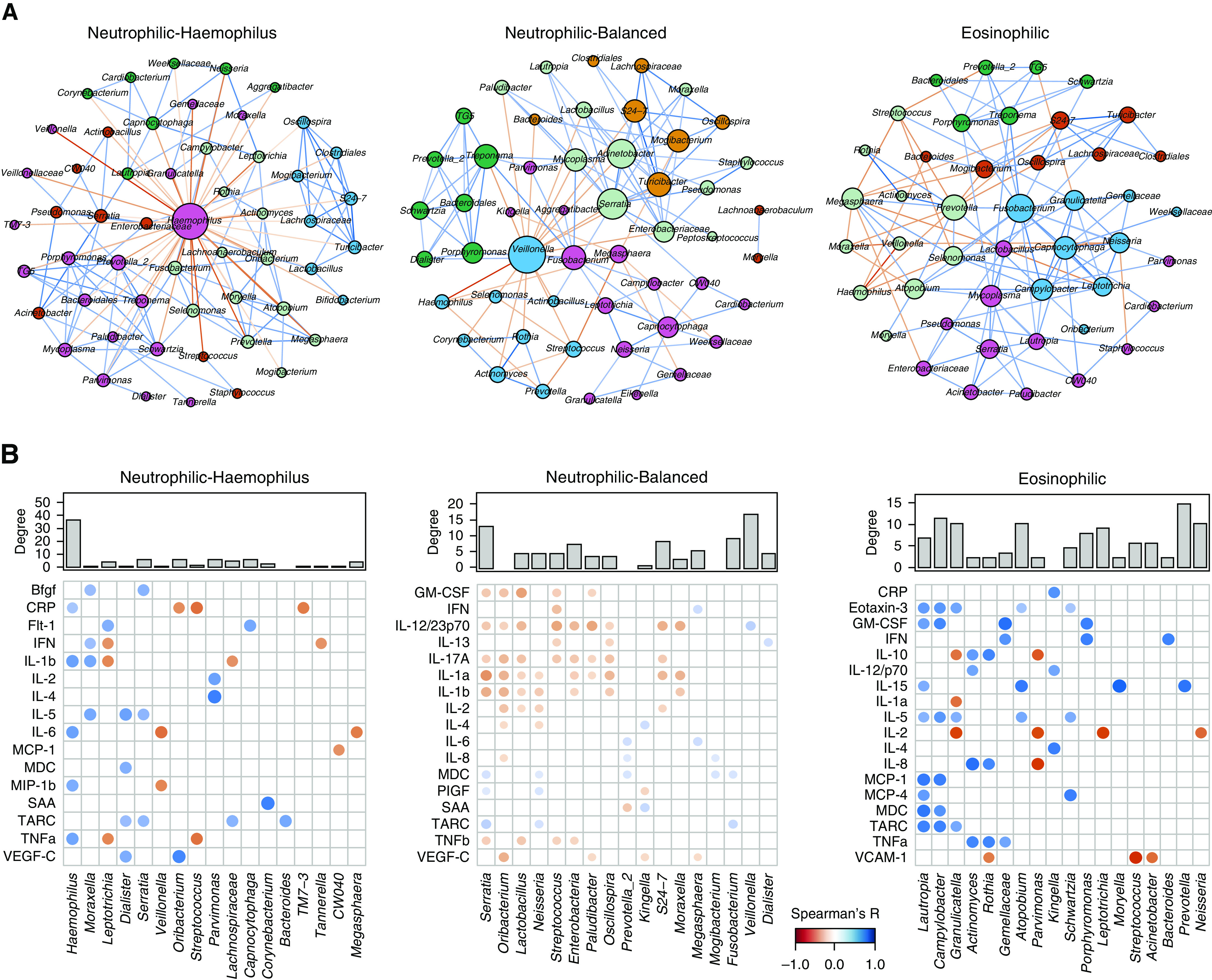
Distinct host–microbiome interactions between inflammatory subtypes. (*A*) The microbiome cooccurrence network for significant correlations between microbiome genera identified by SparCC (Sparse Correlations for Compositional data) in the COPDMAP (Chronic Obstructive Pulmonary Disease [COPD] Medical Research Council/Association of the British Pharmaceutical Industry) and AERIS (Acute Exacerbation and Respiratory Infections in COPD) cohorts. Each node represents a genus. The size of the node is proportional to its degree of connectivity. Nodes were colored by their module assignments by the “Modularity” function on the basis of a Louvain community-detection algorithm implemented in Gephi software (resolution = 1.0). Each edge represents a significant correlation between pairs of nodes (false discovery rate *P* < 0.05). The width of the edge is proportional to the absolute correlation coefficient. Edges were colored on the basis of coexclusion (red) or cooccurrence (blue) relationship. The top 100 positive and negative correlations are shown for display purposes. (*B*) Significant correlations between microbiome genera and sputum mediators using residualized all-against-all correlation by using HAllA (Hierarchical All-against-All association testing) in COPDMAP data. Each dot represents a significant correlation between a microbiome genus and a sputum mediator (false discovery rate *P* < 0.1). The size and color strength of the dot are proportional to the Spearman correlation coefficient. Dots were colored on the basis of negative (red) or positive (blue) correlation. The top 20 positive and negative correlations are shown for display purposes. The degree of connectivity for each genus in the microbiome cooccurrence network (in *A*) was indicated above the microbiome–mediator correlation matrix. SAA = serum amyloid A.

To assess the microbiome community structure across inflammatory subtypes, we performed a cooccurrence network analysis. In the neutrophilic *Haemophilus* subgroup, *Haemophilus* was the predominant hub node exhibiting coexclusive relationships against 35 genera (Figure 5A). In the neutrophilic balanced subgroup, *Veillonella* had the highest degree of connectivity, followed by *Serratia*, *Acinetobacter*, and *Mycoplasma* (Figure 5A). The network in the eosinophilic group was featured by the mutual cooccurrence relationships among *Fusobacterium*, *Granulicatella*, *Capnocytophaga*, and *Campylobacter* that were specifically enriched in this group (Figure 5A).

We further performed an all-against-all correlation analysis between microbiome genera and inflammatory mediators for each subgroup, adjusting for patient demographic cofactors. In the neutrophilic *Haemophilus* subgroup, *Haemophilus* and *Moraxella* had the highest number of correlations with mediators, in particular IL-1β and TNFα (Figure 5B). In the neutrophilic balanced subgroup, there were multiple, moderate, negative correlations of *Serratia*, *Oribacterium*, and *Lactobacillus* with IL-17A, IL-1α, and IL-1β (Figure 5B). In the eosinophilic subgroup, positive correlations were identified between *Lautropia*, *Campylobacter*, and *Granulicatella* and eotaxin-3, IL-5, and TARC (Figure 5B). Analysis of BEAT-COPD revealed overall similar patterns (Figure E18); 83.2% of microbiome–microbiome correlations and 77.6% of microbiome–mediator correlations that were significant in the discovery data set were validated in BEAT-COPD (Tables E6 and E7).

## Discussion

Here, we have shown that the airway microbiome is differentially associated with neutrophilic and eosinophilic COPD. It is well established that neutrophilic inflammation is associated with reduced microbial diversity and increased *Proteobacteria* in asthma and COPD (16, 21, 39, 40). Taylor and colleagues showed that neutrophilic asthma had greater variability in the airway microbiota than eosinophilic asthma (21), which is consistent with our observation. However, the heterogeneity in neutrophilic airway ecology has not been examined in detail, given their relatively small sample size. Using unbiased clustering on over a thousand microbiome samples, we showed that neutrophilic COPD consisted of two main subtypes of airway ecology differentiated by the predominance of *Haemophilus*. Segregating these two subgroups could be important clinically, as they were associated with distinct inflammatory profiles and may justify different therapeutic approaches.

The *Haemophilus*-predominant subgroup had decreased α diversity and enhanced proinflammatory mediators, IL-1β and TNFα, consistent with previous observations in both COPD and asthma (41). This group had high temporal stability, highlighting the persistence of *Haemophilus* colonization in the airways as an important pathogenic entity (42). For this subgroup, bacterial colonization is evident and likely amenable to targeted antimicrobial therapies.

We showed that over half of neutrophilic COPD cases had a balanced microbiome profile. This group had lower sputum IL-1β and TNFα and higher sputum and serum IL-17A. In addition, it was temporally dynamic, and patients in this state at stability were not dominated by typical respiratory pathogens in their microbiota but may be susceptible to the greatest microbiome shifts during exacerbations, perhaps suggesting the need for monitoring airway ecology and pathogen acquisition in particular for this patient subgroup. The role of IL-17A has been increasingly recognized in COPD inflammation (43–46). IL-17A induces SAA, which in turn promotes neutrophilia by increasing IL-17A and Th17-regulating cytokines (i.e., IL-6) (47, 48). Therefore, IL-17A and SAA, both elevated in the neutrophilic balanced subgroup, may form a self-perpetuating axis precipitating neutrophilic inflammation.

Our results suggested, albeit with distinct pathophysiology, that neutrophilic and eosinophilic COPD were temporally interchangeable in some patients, and the neutrophilic balanced subgroup served as an intermediate state between neutrophilic *Haemophilus* and eosinophilic states that were otherwise mutually exclusive. The biology underlying this dynamism is uncertain, but it has been shown that therapeutic blockade of Th2 cytokines enhances Th17 inflammation in asthma (49), suggesting that ICS treatment may contribute to the endotypic switches. Seasonality can be another factor contributing to the observed dynamics of the inflammatory pattern (11). It is important to note that our results are not necessarily contradictory to previous findings on the stability of eosinophilic COPD (11, 50), in that some patients experienced transitions to a mixed granulocytic state or had decreased sputum eosinophilia slightly below a 3% threshold but may still have had eosinophilic inflammation. In our analysis, IL-17A was inversely correlated with the nonpathogenic taxa *Oribacterium* and *Lactobacillus*. Whether patients in this subgroup benefit from anti-IL-17 biologics or a microbiome modulation strategy (i.e., probiotics) warrants further investigation. However, the limited efficacy of targeting IL-17 (51) and IL-23 (52) in contrast to the beneficial effects of macrolide treatment in asthma (53, 54) suggests that targeting dysbiosis is likely preferable in this setting.

We identified microbiota features specifically enriched in eosinophilic over neutrophilic COPD and showed that their enrichments were not simply due to the reciprocal decrease of *Haemophilus* abundance and may not be fully explained by concurrent ICS usage. The role of the airway microbiome in eosinophilic inflammation remains uncertain (55). Taylor and colleagues found modest associations between sputum microbiota and eosinophilic asthma (21), although *Gemella*, *Rothia*, and *Porphyromonas* were enriched in the eosinophilic group, which was consistent with our results as well as with those from Millares and colleagues (56). We showed that *Campylobacter* and *Granulicatella* were associated with eosinophilia both cross-sectionally and longitudinally, indicative of patient endotypic switches and associated with Th2 mediators. The same two genera were also enriched in the gut microbiome of patients with eosinophilic esophagitis in response to allergenic foods (57). *Campylobacter* and *Aggregatibacter* were further shown to be capable of inducing eosinophilic chemotaxis and degranulation (58, 59). Given these results, it is possible that there exists a specific human microbiome population favoring the ecological niches of eosinophilia. Moreover, we showed that monitoring temporal changes of these airway microbiome features tracked patient inflammatory status in real time, which supports the potential need for point-of-care diagnosis using sputum samples and based on microbiome biomarkers.

An important strength of this work is the availability of over 1,700 sputum samples across multiple sites across stability and exacerbations, which, to our knowledge, is by far the largest in a single airway-microbiome study. The results in the combined COPDMAP and AERIS cohorts were subject to cross-validation between sites and independent validation in BEAT-COPD. This analytical strategy ensured the robustness of inflammation-associated microbiome signatures in terms of clinical sites, visits, and sampling procedures. Another strength of the work is the long-term patient follow-up and employment of novel time-series analytical techniques, as compared with previous studies with similar purposes that were mostly cross-sectional (21, 41). The longitudinal analysis raised the confidence for the association of the airway microbiome with eosinophilia and revealed the dynamic pattern of the microbiome in relation to patient inflammatory status.

There are several limitations to this study. First, the samples in this study included both induced and spontaneous sputum, which may have differentially impacted the microbiome profiling. However, previous analyses and our analyses suggested that this impact was nonsignificant (17). Although we have assessed contaminations from reagent controls, the extent of oral microbes in sputum needs to be evaluated further. It also remains uncertain how sputum can recapitulate the ecology in the lower airways, given its inherent mixture of variable elements from the upper and lower airways. Second, the longitudinal sampling remained sparse, which somewhat limited the power of time-series analyses. An intensive and regular participant follow-up would allow a finer-scale examination of the temporal variability of the airway microbiome. Third, a subset of participants in BEAT-COPD and COPDMAP had sputum and serum mediators profiled because of limited sample availability. No other host omics data were characterized, and this omission includes transcriptomics, which may have helped better define Th2 and Th17 inflammation according to gene signatures and immune processes (60). Fourth, all three cohorts are observational, and most patients had moderate to severe disease; therefore, multiple confounders such as ICS treatment or antibiotic treatment are present. Further prospective interventional studies (similar to the study by Segal and colleagues [61] in COPD and the studies by Durack and colleagues [62, 63] in asthma) are required to explicitly understand their effects on the airway microbiome. In addition, the underrepresentation of female participants needs to be considered to assess the generalizability of our findings. Last, our results can only be viewed as associations that are subject to further experimental testing to explore their causality.

In summary, the airway microbiome is differentially implicated in neutrophilic and eosinophilic COPD. The microbiome can stratify neutrophilic COPD into subgroups that justify different therapies. Neutrophilic and eosinophilic COPD are interchangeable in some patients, which is related to composition shifts of the airway microbiome. Monitoring temporal variability of the airway microbiome may capture key changes in patient inflammatory status and assist in therapeutic selection. Results in this study highlight the consideration of the airway microbiome in the inflammatory endotype–based patient management in COPD.
